# Ag and Sn Implications in 3-Polker Coins Forgeries Evidenced by Nondestructive Methods

**DOI:** 10.3390/ma16175809

**Published:** 2023-08-24

**Authors:** Ioan Petean, Gertrud Alexandra Paltinean, Adrian Catalin Taut, Simona Elena Avram, Emanoil Pripon, Lucian Barbu Tudoran, Gheorghe Borodi

**Affiliations:** 1 Faculty of Chemistry and Chemical Engineering, Babes-Bolyai University, 11 Arany Janos Street, 400028 Cluj-Napoca, Romania; 2 Department of Polymer Composites, Institute of Chemistry “Raluca Ripan”, Babes-Bolyai University, 30 Fantanele Street, 400294 Cluj-Napoca, Romania; 3 Applied Electronics Department, Technical University of Cluj-Napoca, 400027 Cluj-Napoca, Romania; 4 Faculty of Materials and Environmental Engineering, Technical University of Cluj-Napoca, 103-105 Muncii Bd., 400641 Cluj-Napoca, Romania; 5 Zalau County Museum of History and Art, 9 Unirii Street, 450042 Zalau, Romania; 6 Faculty of Biology and Geology, Babes-Bolyai University, 44 Gheorghe Bilaşcu Street, 400015 Cluj-Napoca, Romania; 7 National Institute for Research and Development of Isotopic and Molecular Technologies, 65-103 Donath Street, 400293 Cluj-Napoca, Romania

**Keywords:** forged 3-Polker microstructure, Ag-plated copper, Sn-plated copper, SEM–EDX, XRD

## Abstract

Several forged 3-Polker coins have been reported in historical sources on the financial crisis that occurred between 1619 and 1623 at the start of the 30-year-long war. Supposedly, belligerent countries forged other countries’ coins which were then used for external payments as a war strategy. Thus, a lot of 3-Polker coins (e.g., Sigismund-III-type) were forged, and the markets became flooded with poor currency. In the present day, these pre-modern forgeries are rare archeological findings. Only five forged 3-Polker coins randomly found in Transylvania were available for the current study. There are deeper implications of silver and tin in the forgery techniques that need to be considered. Thus, the forged 3-Polker coins were investigated via nondestructive methods: SEM microscopy coupled with EDS elemental spectroscopy for complex microstructural characterization and XRD for phase identification. Three distinct types of forgery methods were identified: the amalgam method is the first used for copper blank silvering (1620), and immersion in melted silver (1621) is the second one. Both methods were used to forge coins with proper legends and inscriptions. The third method is the tin plating of copper coins (with corrupted legend and altered design) (1622, 1623, and 1624). The EDS investigation revealed Hg traces inside the compact silver crusts for the first type and the elongated silver crystallites in the immersion direction, which are well-attached to the copper core for the second type. The third forgery type has a rich tin plating with the superficial formation of Cu_6_Sn_5_ compound that assures a good resistance of the coating layer. Therefore, this type should have been easily recognized as fake by traders, while the first two types require proper weighing and margin clipping to ensure their quality.

## 1. Introduction

The 30-year-long war began in 1618 in the Holy Roman Empire during the reign of Emperor Ferdinand II due to the internal friction between Protestants and Catholics, which was reminiscent of the religious wars in central Europe [1,2,3]. German members of the Holy Roman Empire (Protestants) generated internal revolts against the Catholic emperor. These were supported by external help in the first phase. Prince Gabriel Bethlen of Transylvania joined the war on the side of Ferdinand II. Thus, he received the Duchy of Oppelin and Ratibor from the Holy Roman Empire [4,5].

An economic strategy of forging coin types used by nonbelligerent countries was implemented at the beginning of the war, intended for external payments as far as possible from their own territories. The strategy only worked at the beginning of the war, and eventually, the forged coins returned to the internal market, generating a severe crisis. Silver coins were kept and hoarded while the forged ones were kept in circulation, generating a huge rise in inflation. The price stabilization attempts were useless, with the prices being formed on the market for demands such as 72 Kreutzer for a Reichsthaller in 1600; 124 Kreutzer for a Reichsthaller in 1619; 390 Kreutzer for a Reichsthaller in 1621; and over 600 Kreutzer for a Reichsthaller in 1622 [6,7]. Traders and official tax collectors began testing each coin by weight and clipping its margin to observe the interior core; thus, the crisis gained the name of Kipper und Wipperzeit [6,7].

Contemporary Transylvanian sources mentioned a significant copper-rich 3-Polker afflux from the Duchy of Oppelin and Ratibor as a consequence of the Kipper und Wipperzeit crisis [8]. The Transylvanian Diet (legislative forum) held in May 1622 at Cluj decided that the official exchange rate was four silver denars for a silver 3-Polker coin and two silver denars for suspicious coins [9,10]. An intensive debate has occurred among historians due to the lack of detail in medieval sources: some of them considered that the official Poland 3-Polker was strongly debased because it was rich in copper, while others supposed that the copper-rich 3-Polker mentioned in medieval sources might be the forged coins related to Kipper und Wipperzeit crisis. Our previous research evidenced that the official Poland 3-Polker issues maintain a fair silver title of good coins and therefore are hoarded [10]. Thus, the forged 3-Polkers are the copper-rich coins mentioned by medieval sources. But where are they now? It is hard to find them using the usual archeological methods, and they are typically found only by random chance. The explanation is that Gabriel Bethlen, a Transylvanian Prince, decided on an internal currency stabilization that issued new coins stricken with good silver and withdrew the forged coins from circulation at the rate of one good coin for five forged coins. The withdrawn forged coins were melted, and the resulting copper was used for other purposes [8,11]. The infusion of high-silver title coins refreshed the economic activities and stabilized the local market. However, several forged coins remain to the present day and occasionally appear in the ground or in forgotten places.

The silver plating of copper cores has been used for coin forgery since antiquity within Greek Kingdoms and the Roman Empire [12,13]. The Roman coin depreciation, along with the consequent inflation, led to the official practice of issuing copper denars and antoninians plated with a thin silver foil [14]. Two basic techniques were used. The most effective one was the immersion of a copper blank into melted silver. Thus, a local melting occurs between the copper blank and silver, forming an intermediary eutectic zone and a pure silver layer on the exterior [12,14]. Another method was to attach a thin silver foil via thermo-mechanic treatment during hot coin hammering, with the silver foil being attached to the copper blank via a thin layer of Ag-Cu eutectic formed by the soldering process. This is similar to the Sheffield plating technique that is used in the present day [15]. These ancient techniques were currently used in medieval times, along with some unusual methods such as the application of a silver amalgam on the copper blank followed by the cold hammering of the coin [16,17]. It was supposed that a copper blank with a properly cleaned and degreased surface was passed through silver amalgam. Superficial tension between the copper blank and the amalgam allows for a thin-layer deposit on its surface. Mercury evaporates after extraction from the amalgam, leaving a thin layer of high-purity silver [18,19].

The data are scarce regarding tin-plated medieval counterfeits, but it seems that blank copper was often immersed in molten tin before coin hammering [16]. Thus, it is very interesting to conduct a study regarding such counterfeits that most likely were widely circulated during the Kipper und Wipperzeit crisis.

Several forged 3-Polkers within the Zalau Museum of History and Art were selected for the current investigation. The aim is to find out the possible forging techniques and make connections with medieval and pre-modern historical sources. Generally, the artifact’s elemental composition is investigated by XPS [12,19] and XRF [16], but the result strongly depends on the surface-cleaning quality. Furthermore, these methods did not allow for a direct correlation to the investigated area microstructure. The SEM–EDX elemental investigation has the advantage of being able to properly choose the investigation site on the artifact surface, allowing us to avoid dirt and corroded spots [20,21]. Another advantage is being able to couple them with high-resolution images of the microstructure.

SEM–EDX elemental maps show the element’s distribution within the observed microstructure. Each element has its own color, and thus the alloy constituents and phases appear in specific and distinct nuances [22,23]. Thus, the second aim of the present research is to employ SEM–EDS elemental maps for classical metallographic analysis avoidance (which is destructive due to the grinding and polishing steps regarding sample preparation).

There are some limitations of the research due to the nondestructive methods and the low number of forged coin samples used that are considered for the resulting discussion and allow for the drawing of relevant conclusions, which opens a new path to continue research involving materials specialists and historians.

## 2. Materials and Methods

### 2.1. Samples Description

**Figure 1:**
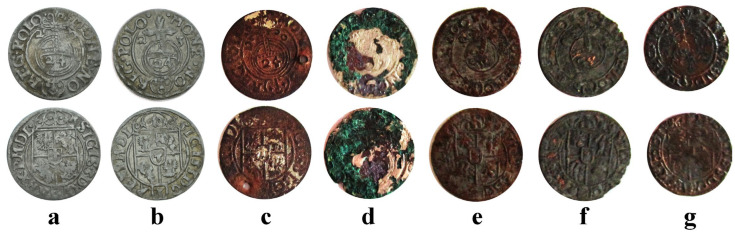
The investigated 3-Polker coins. Official Polish minted: (**a**) 1620 and (**b**) 1623; forged: (**c**) 1620, (**d**) 1621, (**e**) 1622, (**f**) 1623, and (**g**) 1624.

The samples investigated in the current study are a type of 3-Polker coins like the issues of Sigismund III, King of Poland (Figure 1). The first two coins are original genuine Polish issues from 1620 (Figure 1a) and 1623 (Figure 1b). Both original coins were minted at Bydgoszcz (Bromberg) Mint. The legend on the obverse shows the following inscription, SIGIS(mundus) 3 D(ei) G(ratia) REX P(oloniae) M(agnus) D(ux) L(ithuaniae), meaning Sigismundus the third by God mercy King of Poland and Grand Duke of Lithuania. The inscription surrounds the heraldic emblem of the Kingdom of Poland. The legend on the reverse faces the following inscription, MONE(ta) NO(va) REG(ni) POLO(niae), meaning new coin of Polish Kingdom, surrounding a cross orb with the coin value marked on 24—meaning that 24 pieces are equivalent of a standard taller. The minting year numerals are placed below horizontal arms of the cross (e.g., 2 and 0 for 1620).

The forged 3-Polker coins are presented in Figure 1c–g. The ones in Figure 1c,d present the correct legends and design, and only some characters are slightly bigger than on the original coins. These forgeries are from 1620 (Figure 1c) and 1621 (Figure 1d). The following forged coins (Figure 1e–g) have corrupted legends, being almost meaningless, but it imitates the original inscriptions (it worked in those ages because most people could not read); the numeral 24 in cross-orb is figured by naive inscription similar to “GS”.

The investigated coins belong to the collection of the Zalau Museum of Art and History, Salaj, Romania, and were subjected only to nondestructive investigation without any alteration of their consistency. 

Artifact cleaning is one of the most important steps regarding their investigation. Therefore, all involved coins were properly cleaned according to the museum procedures using a solution of Complexon 3 EDTA (Sigma-Aldrich, Darmstadt, Germany) with concentration of 3.72%. Each coin was immersed in this solution at 70 °C for 30 min, followed by intensive washing with bi-distilled water. This procedure allows for a gradual cleaning of the surface of the sample, assuring a lot of clean micro-sites. Some of the cleaned sites still contain very small influences of the corrosion layer that acts as a genuine chemical etching, enhancing the microstructural observation without using an etching reactive.

### 2.2. Investigation Methods

Investigation protocol requires only nondestructive methods to preserve the samples’ integrity and their museum value. Therefore, we chose Scanning Electron Microscopy (SEM) for the microstructural observation and elemental spectroscopy, including distribution maps on the proper microscopic sites on the sample’s surface. It facilitates avoiding areas where local corrosion remains. The elemental composition of the sample’s microstructure was correlated with the crystalline phases occurring in the involved alloys being identified by X-ray diffraction. Both elemental composition and crystalline phases were further discussed regarding the phase diagrams.

SEM investigation was effectuated using a Hitachi SU8230 microscope with an Energy Dispersive Spectroscopy (EDS) detector X-Max 1160 EDX (Oxford Instruments, Singapore). It was operated in high vacuum mode at an acceleration voltage of 30 kV. This was followed by secondary electron images for a high-resolution view of the microstructure and EDX spectroscopy associated with the elemental distribution maps. At least 3 different macroscopic sites were investigated for each sample.

The X-ray diffraction (XRD) was effectuated using a Bruker (Billerica, MA, USA) D8 Advance diffractometer with CuKα1 monochromatic radiation (λ = 1.54056 Å). XRD patterns were recorded from 30 to 100 degree 2 theta for the original 3-Polkers and from 20 to 100 degree 2 theta for the forged coins. The patterns were registered at a speed of 1 degree per minute. Peaks were identified using the Match 1.0 software equipped with the PDF 2.0 XRD database provided by Crystal Impact Company, Bonn, Germany.

### 2.3. Statistical Analysis

The elemental composition of the investigated samples was statistically processed using Anova test with Tukey post hoc test with a significance level of 0.05. The statistical analysis was performed with Microcal Origin 2019b (Microcal Company, Northampton, MA, USA). The tested statistical populations with *p* < 0.05 present significant differences, while *p* > 0.05 shows statistical similarities.

## 3. Results

### 3.1. Original 3-Polkers

The background of the current research relies on the investigation of the original 3-Polker coins issued by Sigismund III, King of Poland, in 1619–1627, which was extensively explored in one of our previous studies [10]. It was evidenced that rich silver coins issued between 1619 and 1620 have a silver title over 85%. The financial crisis collaterally affected Poland, causing a slight depreciation of the silver title below 80% in 1621–1625, but it was restored to normal value after 1626 by the new regulations introduced by the new crown treasurer Hermann Ligenza.

**Figure 2:**
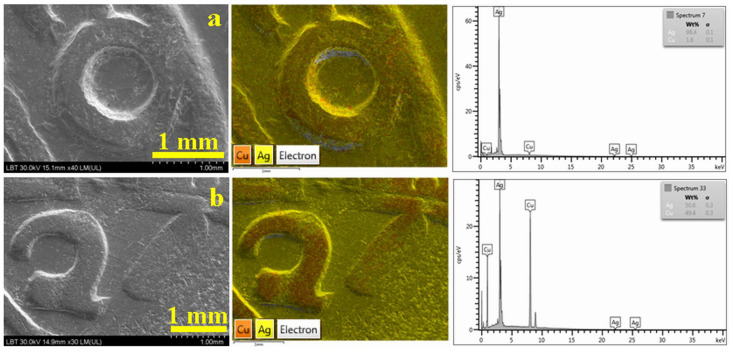
SEM images with corresponding EDS map and spectrum of the official Polish minted 3-Polkers: (**a**) 1620 and (**b**) 1623.

Thus, the most relevant original coins for present research are a 3-Polker issued in 1620 at Bydgoszcz mint as a reference for a high-silver title coin (Figure 2a) and a depreciated silver title one issued in 1623 (Figure 2b). Both original coins bear the mintmark of crown treasurer Nicolas Danilowicz.

The original coin issued in 1620 presents sharp details of the impression with significant wear marks on the top of the inscriptions (Figure 2a). The elemental distribution map shows a uniform distribution of Ag and Cu, proving the coin homogeneity. The EDS spectrum registered on the coin surface reveals a weight composition of 98.4% Ag and 1.6% Cu of hypoeutectic type proving the high title of the coin.

The original 3-Polker issued in 1623 presents a nice silvery surface that would be considered of high title (see its uniform macroscopic aspect in Figure 1b), but the worn marks on the top of the inscriptions reveal the depreciated title of the alloy inside the coin. It appears as reddish areas in the elemental maps within Figure 2b. The EDS spectrum registered on the worn marks’ areas reveals a weight composition of 50.6% Ag and 49.4% Cu of hypereutectic type that corresponds to the depreciated title.

**Figure 3:**
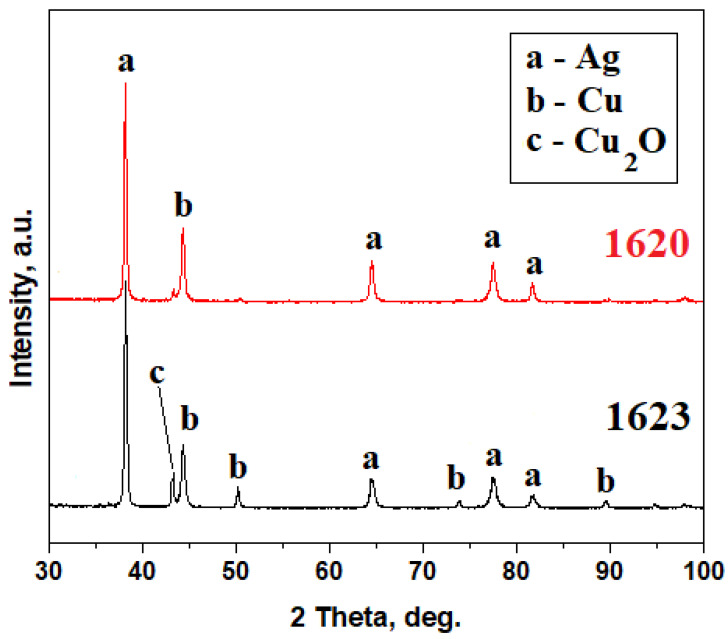
XRD patterns of the official Polish 3-Polkers minted in 1620 and 1623. Indexing files: Ag PDF 65-2871 and Cu PDF 89-2838.

Both original coins were subjected to the X-ray diffraction (XRD) investigation; the obtained patterns are presented in Figure 3. The XRD pattern for the original from 1620 is dominated by the Ag peaks, which are very well developed, and a single copper peak is visible (Figure 3); thus, applying the RIR method [10,24,25] results in a weight composition of 93.17% Ag and 6.83% Cu, fact in good agreement with EDS observation.

The original coin issued in 1623 presents a more complex XRD pattern (Figure 3) dominated by silver peaks, which are closely followed by copper peaks and small traces of cuprite. Thus, applying the RIR method results in a weight composition of about 57.22% Ag and 42.78% Cu. It is situated in the hypereutectoid domain of the Ag-Cu binary system and completely agrees with the observed elemental composition.

### 3.2. Forged 3-Polkers

**Figure 4:**
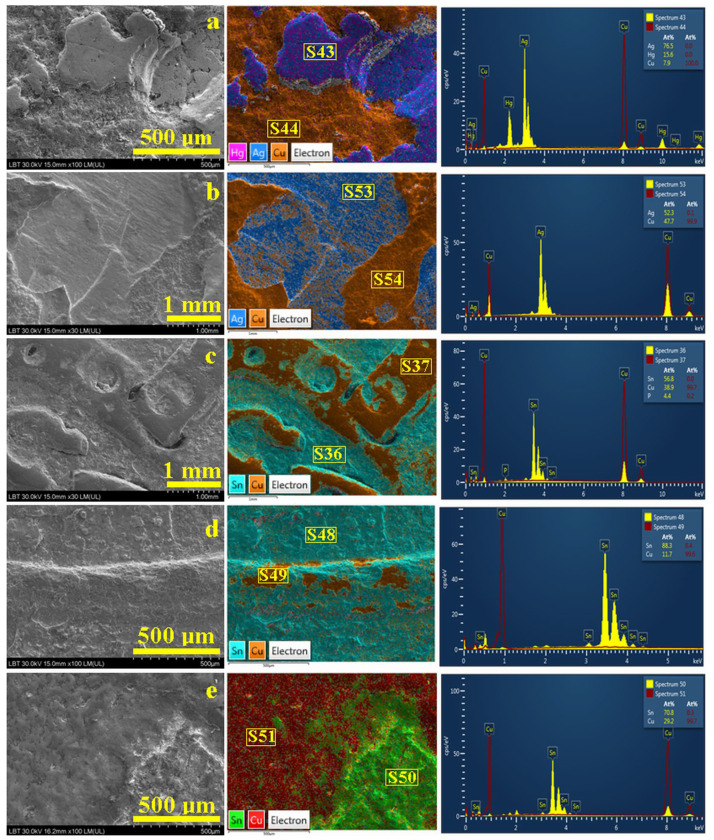
SEM images with corresponding EDS map and spectrum of the forged 3-Polker coins: (**a**) 1620, (**b**) 1621, (**c**) 1622, (**d**) 1623, and (**e**) 1624.

All investigated forged 3-Polker coins feature copper bases and different coatings with silvery aspects, as observed in Figure 1. Historical information contains a lot of doubts regarding these counterfeits. Therefore, SEM–EDX investigation of their surface at low magnification was necessary for the samples’ overview characterization (Figure 4). Electron images were used for surface morphology monitoring, and the corresponding EDS maps were used for elemental tracing of the coating remains. Individual spectra were registered both on the copper substrate and coating areas.

The forged coin from 1620 shows a deeply eroded surface with some traces of silvery foil; thus, large scales ranging from 30 μm to over 600 μm are visible in Figure 4a. The elemental map reveals that the larger scales colored in blue nuance are, in fact, silver areas containing traces of mercury (pink dots). EDS spectrum S43 reveals an atomic percent composition of 76.5% Ag, 7.9% Cu, and 15.6% Hg. The composition belongs to a hypoeutectic silver alloy according to the Ag-Cu binary system phase diagram. The significant mercury content indicates that the coating was deposited by the amalgamation method.

The copper area appeared in a brown nuance and was investigated by EDS spectrum S44 revealing a composition of 100% Cu belonging to the copper substrate of the coin.

The forged coin issued in 1621 presents a different surface type showing polyhedral domains ranging between 0.5–1.5 mm, with the upper ones being relatively smooth and the lower ones being significantly corrugated (Figure 4b). The surprise arises on the corresponding elemental map where the smooth domains feature a eutectic aspect based on the lamellar-grained structure of Ag (blue) and Cu (brown) grains. S53 spectrum evidence the atomic elemental composition of 52.3% Ag and 47.7% Cu being situated closely to the eutectic composition in the Ag-Cu phase diagram. S54 taken on the brown areas evidence an elemental composition of 99.9% Cu. The eutectic presence over the copper blank indicates immersion into the melted silver as a coating method.

Counterfeited coins from 1622, 1623, and 1624 have great macroscopic similitude, such as a silvery coating with a gray tarnish and significant excoriations exposing parts of the copper core. Thus, SEM imaging reveals a slightly corrugated surface of the coating remains and a smooth surface of the copper core (Figure 4c–e). Fact is sustained by the aspect of the elemental maps where smoother areas are colored in brown nuance specific for pure copper, and rougher ones have turquoise and green shaded areas belonging to the Sn-rich coating remains. The EDS spectra were taken from each of the representative areas on these coins, and the obtained values are centralized in Table 1.

The elemental composition of the coating corresponds to a bronze alloy very rich in tin content according to the Cu-Sn phase diagram, especially for the forgery from 1622. The coating layers of the forged coins from 1623 and 1624 contain more tin than the previous one. These areas are corrugated both due to circulation and further corrosion under resting soil conditions. 

Also, it is amazing how well-preserved the worn marks on the copper-rich areas are, proving that forged coins were in circulation for a long time until their disposal or loss. A small amount of tin detected on the copper substrate for 1623 and 1624 coins indicates a local alloying of the copper blank surface during immersion into the tin-rich melted bath. Also, a progressive dissolution of copper from the immersed blanks might lead to an increase in the copper amount of the molten tin bath generating an uneven process that is less controlled in terms of composition.

**Figure 5:**
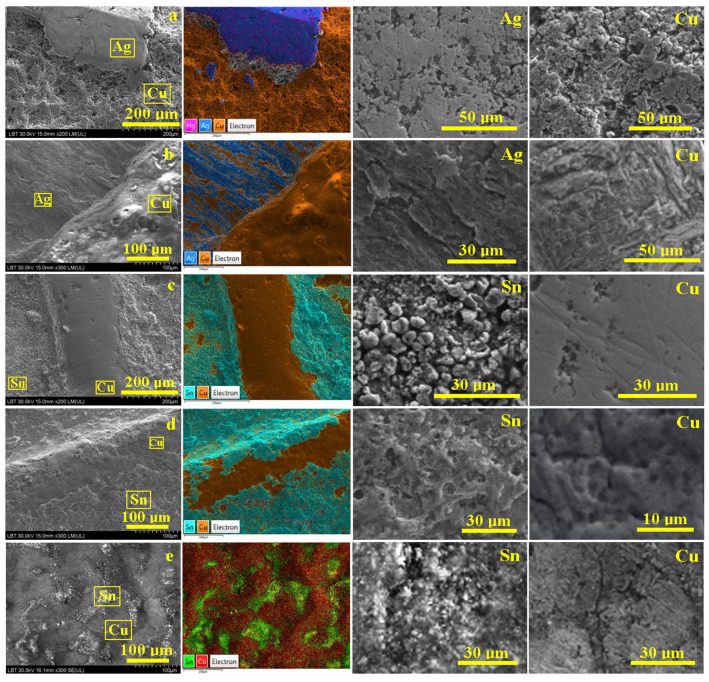
SEM images with microstructural details and their corresponding EDS map of the forged 3-Polker coins: (**a**) 1620, (**b**) 1621, (**c**) 1622, (**d**) 1623, and (**e**) 1624.

Microstructural details are necessary for further observation of the metallurgical aspects regarding how forgeries were performed. It is mandatory to observe the coating adhesion mode on the substrate and the grains interlocking. It was possible by a slow inclination of the sample of about 30° regarding the SEM electron beam. Therefore, high-magnification SEM images were taken on the domain’s interface border (Figure 5). 

The copper substrate of the 1620 forged 3-Polker has a refined grain microstructure with rounded shapes and sizes of about 10–25 μm with a relatively corroded aspect, as seen on the right side of Figure 5a. The border of the silver coating is sharp and well-defined, indicating a lack of diffusion interlayer, and is situated from the left to upper corners of the image. The silver coating is very compact and smooth with a typical α grain structure (α-rich silver phase of Ag-Cu diagram) with polyhedral shapes and sizes ranging from 20 to 30 μm. EDS map shows that mercury traces are uniformly distributed within the silver foil, indicating a physical attachment of the silver to the copper blank due to Hg evaporation during amalgam pellicle solidification.

The microstructure of the 1621 forged 3-Polker is more complex, as observed in Figure 5b. The left side of the image features the silver coating, and the microstructure constituents’ grains are very visible: blue shade ones are Ag-Cu eutectic and are strongly mixed with β copper grains. Both grain types are elongated, indicating the immersion direction, and have a length and width of about 30 μm and 15 μm, respectively, while eutectic and copper grains are slightly smaller, with a length and width of about 25 μm and 10 μm, respectively. This indicates a local melting of the copper blank surface at the contact with molten silver eutectic composition generating a diffusion interlayer. Thus, the silver coating proves to be very resistant and gives the appearance of a high-title silver coin. The copper substrate, left side of Figure 5b, has large grains of about 50 μm with a twinned intra-grain structure as observed in the provided microstructural detail.

All tinned forged coins present a slightly corrugated microstructure of the coating remains with small crystallites of about 5–15 μm as observed in Figure 5c,d, and e on the Sn microstructural details. The corrugated aspect is caused by resting on the ground for about 400 years until they were randomly discovered and handed to the museum. The immersion method for copper tin plating supposes the formation of an intermediary layer which is slightly visible in Figure 5c,d, where the delimitation border appears sharp and well-defined. However, the forgery from 1624 reveals a small intermediary zone formed between the tin layer and copper core with very small grains of about 3–10 μm, as observed in Figure 5e. The copper blank areas are smooth and present large polyhedral copper crystallites of about 20–50 μm (microstructural details for Cu within Figure 5c–e), a fact related to advanced annealing during immersion into the melted tin bath.

**Figure 6:**
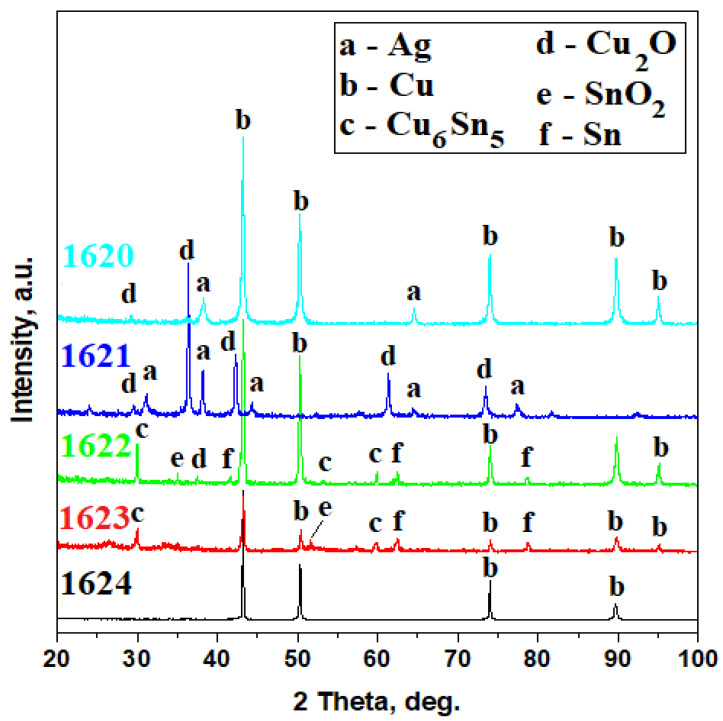
XRD patterns of the forged 3-Polker coins issued in 1620, 1621, 1622, 1623, and 1624. Indexing files: Ag PDF 65-2871; Cu PDF 89-2838; Cu_6_Sn_5_ PDF 78-1575; Cu_2_O PDF 78-2076; SnO_2_ PDF 77-0452; and Sn PDF 05-0930.

Microstructural constituents of the forged 3-Polker coins were correlated with the phase compositions evidenced by the XRD investigation (Figure 6). The coin from 1620 presents a diffraction pattern with very well-developed peaks belonging to pure copper and only two small pure silver peaks (Figure 6). It corresponds to the general aspect of the coin, which presents only a few traces of silver foil.

The other silvered coin generates a more complex XRD pattern (Figure 6), where the peaks are very well developed with a narrow shape that corresponds to a highly crystalline state of the material. The dominant peak belongs to the cuprite (Cu_2_O) due to the large, corroded areas on the surface of the coin, but fortunately, there appear significantly relevant peaks of silver that sustain the microstructural observation.

XRD pattern in Figure 6 reveals the phase composition of the forged coin from 1622. It is based on the copper intense peaks that are accompanied by well-developed peaks belonging to the Cu_6_Sn_5_ intermetallic compound that occurs on the tin-rich area of the Cu–Sn binary system and some peaks of pure Sn. There are also appearing some small peaks belonging to cassiterite SnO_2_. A similar observation was found for the forged coins from 1623 (Figure 6). Copper substrate proves to be more visible in the case of the forged coins from 1624, being visible only in the pure copper peaks (Figure 6).

### 3.3. Statistical Results

**Figure 7:**
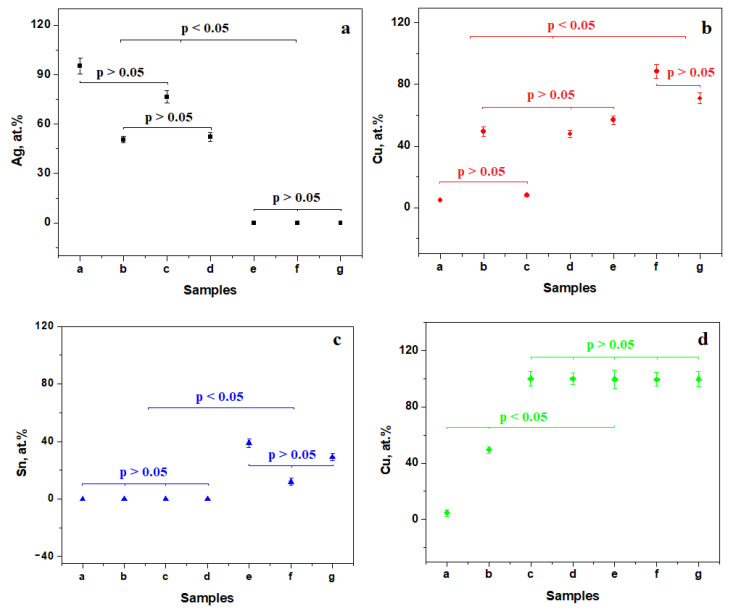
Statistical results for the elements found in the sample surface layer: (**a**) Ag, (**b**) Cu, (**c**) Sn, and (**d**) Cu from the forged coins substrate.

Elements such as Ag, Cu, and Sn from the coin surface layer were considered as statistical populations and further processed. The results are centralized in Figure 7. Three statistical populations were found for the Ag content. The high-silver title coin has statistical similarities with the forged coin from 1620, forming the first group (Figure 7a). The second statistical group is formed by the low-silver title coin and the forged coin from 1621. Forged coins from 1622, 1623, and 1624 have no silver on their surface and form the third statistical group. A significant statistical difference occurs among those three groups indicating three classes of forged coins. Similar statistical aspects are observed for the copper amount from the coin surface (Figure 7b).

Sn amount statistics evidence only two statistical groups (Figure 7c). The first group gathered all samples that contained silver without tin, and the second group the samples containing tin without silver. This situation sustains the third forgery method evidenced by the microstructural investigation.

We admit that the silver coins in Figure 1a,b have the same composition on their surface as well as in the coin core due to the elemental analysis performed on the areas of maximum wear on the top of the inscription. Thus, the copper content of the coins’ substrate is followed in Figure 7d. There are three statistical populations. The first two correspond to the copper content within the good coins. The third population belongs to the forged coins copper substrate. 

## 4. Discussion

The investigation of the original 3-Polker coins issued by Polish mints in 1620 and 1623 confirms the conclusions of our previous study [10] that evidence the increased silver title for these coins up to 1620 and sustained slight depreciation of the title beginning in 1621. This depreciation was a little collateral consequence of the financial crisis that developed under the Holy Roman Empire and emergent territories such as the Transylvania Principate. This observation is sustained by a large number of original 3-Polkers found in the hoards discovered in Transylvania, such as the Salajeni Hoard [26], Verveghiu Hoard [27], Zalau Hoard No. 4 [28], Mineu Hoard [29], and Aghires Hoard [30]. No forged coin was identified among all the hoards mentioned. Therefore, the high-silver title coins were hoarded by the traders while the suspicious and forged coins were passed into circulation.

The hoard contents are silent witnesses of the dark side of the Kipper und Wipperzeit crisis: each coin was weighed and clipped on the side for their content verification [6,7]. Thus, qualitative forgeries were required for successfully tricking the traders. Our results reveal that the incipient forgeries related to the war strategy were manufactured in a specialized facility, a fact sustained by the inscription quality and coin design for the ones issued in 1620 (Figure 1c) and 1621 (Figure 1d). 

The first one is silvered by the amalgam method, a fact proved by the mercury traces identified in the silver foil remains. It is rather a physical method of silver attachment than a chemical one which presumes the immersion of the copper blank into the silver amalgam. Superficial tension has a key role in preventing excessive contact of the amalgam with the metallic surface [31,32]. Therefore, silver deposit on the copper blank results in a thin silver foil without much adherence. Thus, the deposited foil adherence was increased by the coin hammering, which presses the fine grains resulting after Hg evaporation into a compact structure embedded into the copper grains of the substrate, a fact proved by the microstructural aspects observed in Figure 5a. Cold pressing of the thin silver foil is sustained by the lack of Ag-twined grains, which are indicators of tin foil recrystallization [33,34]. A clear mark of the “Kipper” element of the crisis is the hole pierced into the forged coin from 1620 that clearly reveals its copper core. This forgery method is sustained by statistical analysis and clearly shows the silvered layer resemblance to the very good silver title coins.

The second qualitative forgery is the coin from 1621. The obtained results prove that they were produced by the immersion of the copper blank into the molten silver alloy of eutectic composition (60% at. Ag; 40% at. Cu) that partially melts the surface of the copper blank, forming a coherent adhesion interlayer. The eutectic lamellar grains formation [35,36] respects the direction of blank immersion into the molten composition. Thereafter, the coin was cold hammered, consolidating the silver coating adhesion through the diffusion interlayer. The final silver coating is very resistant and perfectly imitates the aspect of the original 3-Polker, but the copper core makes them lighter and consequently would be rejected by the “Wipperzeit” test. As a consequence, this coin was not found in a hoard, but it was a random finding during some civil excavations. The resemblance of the silvered layer to the depreciated silver coins is sustained by statistical analysis.

Tinned forged 3-Polkers from 1622, 1263, and 1624 are unusual coins due to their corrupt legends and value inscription. It looks like they were produced by some unlettered counterfeiters, but there are very interesting aspects regarding production techniques that were unavailable to people without proper knowledge. The obtained results indicate the presence of Cu_6_Sn_5_ intermetallic compound corresponding to the concentration of 44.6 Sn % at.% from the Cu-Sn binary phase diagram. It indicates that the molten bath contains a tinrich bronze instead of pure tin [37,38]. It facilitates the formation of a small adhesion interlayer to the copper blank, which assures a more resistant coating, a fact reported in the literature for high-tin bronze artifacts [39,40]. Statistical analysis clearly shows that these forgeries are a distinct group.

This has direct implications for reconsidering these forgeries. Apparently, they were effectuated by some illiterate villains, but in fact, the high level of metallurgical skill besides their manufacturing process indicates that they were issued in specialized facilities, and the corrupt design might be a smart disguise of their origin. It looks like they were prepared for payments in oriental territories where Latin scripts were illegible, and thus the incorrect legend was not relevant. Their issuing after 1622 is another inconsistency because the forging strategy was officially abandoned, but it was hard extinguishing it from the current practice. The official data in Transylvania show that all forged coins were withdrawn from circulation at a rate of one good coin for five forged and the withdrawn coins were melted down. Unfortunately, there is no material proof that it is so. Only the rarity of the forged 3-Polkers findings agrees with the medieval source.

The present research has certain limitations due to the low sample number and nondestructive method used for investigation, which does not allow coin sectioning. The small sample number was compensated by an adequate number of measurements performed on each sample, allowing statistical analysis. The relevant information was acquired from the surface of the coins by a smart orientation of the observation field during the SEM–EDX investigation. Overall, the obtained results properly illustrate the Ag and Sn implications in the forgeries process, being useful for historians but also for the materials scientists involved in archaeometric investigation.

## 5. Conclusions

The present research focused on forged 3-Polker coins found in Transylvania, revealing three types of counterfeits sustained by the statistical analysis:

− Copper core plated with a thin silver foil via amalgamation method, evidenced on the forged coins from 1620;
− Copper core plated with a relatively thick silver layer deposited by immersion into an Ag-Cu eutectic molten bath, evidenced by the forged coin from 1621;
− Copper core plated with high-tin bronze bases on the bi-phase structure of Cu_6_Sn_5_ intermetallic compound and pure Sn grains, evidenced by the forged coins from 1622, 1623, and 1624.

The first two types are the product of the Kipper and Wipperzeit financial crisis strategies, while the last type, based on Sn coating instead of silver, might be attributed to some occult economic operation uncovered by the official reports within the historical sources. Thus, the Ag and Sn implications in the forgery process were successfully assessed.

SEM–EDX investigation effectuated in a smart manner provides high-quality images and elemental maps of the plating layer–copper substrate junction allowing a proper microstructural characterization. Thus, coin sectioning and metallographic investigation are avoided. Proper adjustment of the sample angle regarding the electron beam assures an optimal view of the desired microstructures.

## Figures and Tables

**Table 1:** Elemental composition of tinned forged coins.

Forged Coin Year	EDS Spectrum No.	Elemental Composition at. %		
		Sn	Cu	P
Coating areas				
1622	36	56.8	38.9	4.4
1623	48	88.3	11.7	-
1624	50	70.8	29.2	-
Copper substrate				
1622	37	-	99.7	0.2
1623	49	0.4	99.6	-
1624	51	0.3	99.7	

## Data Availability

Data are available on request from the corresponding author.

## References

[1] McDougall W.A. (2020). The Myth of the Secular: Religion, War, and Politics in the Twentieth Century. Orbis.

[2] Schaff F.S.F. (2022). Warfare and Economic Inequality: Evidence from Preindustrial Germany (c. 1400–1800). Explor. Econ. Hist..

[3] Eyerman R. (2023). Race in the Culture Wars. Religions.

[4] Ludwig B. (2021). Baroque Origins of the Greenery of Urban Interiors in Lower Silesia and the Border Areas of the Former Neumark and Lusatia. Sustainability.

[5] Buza J., Tamas F., Emoke G. (2021). Inflációs Pénzforgalom Törvénye Segédlettel; az 1622. évi 77. törvénycikk (Inflationary money circulation is legal with assistance; Article 77 of 1622). A Rendtartó Történész Tanulmányok Imreh István Születésének Századik Évfordulójára.

[6] Karaman K.K., Pamuk Ş., Yıldırım-Karaman S. (2020). Money and monetary stability in Europe, 1300–1914. J. Monet. Econ..

[7] Boubaker H., Cunado J., Gil-Alana L.A., Gupta R. (2020). Global crises and gold as a safe haven: Evidence from over seven and a half centuries of data. Phys. A Stat. Mech. Its Appl..

[8] Mihailescu S.G. (1993). Nagy SzabóFerencz of Târgu Mureș Memorial (1580–1658). Translation, Notes and Introductive Study.

[9] Sandor S. (1882). Erdélyi Országgűlési Emlékek (Monumenta Comitialia Regni Transylvaniae; Monuments of the Transylvania Shire).

[10] Petean I., Paltinean G.A., Pripon E., Borodi G., Barbu Tudoran L. (2022). Silver Depreciation in 3-Polker Coins Issued during 1619–1627 by Sigismund III Vasa King of Poland. Materials.

[11] Kiritescu C. (1964). Sistemul Bănesc al Leului și Precursorii săi.

[12] Ingo G.M., Riccucci C., Faraldi F., Pascucci M., Messina E., Fierro G., Di Carlo G. (2017). Roman sophisticated surface modification methods to manufacture silver counterfeited coins. Appl. Surf. Sci..

[13] Constantinescu B., Săşianu A., Bugoi R. (2003). Adulterations in first century BC: The case of Greek silver drachmae analyzed by X-ray methods. Spectrochim. Acta Part B At. Spectrosc..

[14] Pense A.W. (1992). The decline and fall of the roman denarius. Mater. Charact..

[15] Zwicker U., Oddy A., La Niece S., La Niece S., Craddock P. (1993). Roman techniques of manufacturing silver-plated coins. Metal Plating and Patination.

[16] Hložek M., Trojek T. (2017). Silver and tin plating as medieval techniques of producing counterfeit coins and their identification by means of micro-XRF. Radiat. Phys. Chem..

[17] Beck L. (2022). Ion Beam Analysis and ^14^C Accelerator Mass Spectroscopy to Identify Ancient and Recent Art Forgeries. Physics.

[18] Fajfar H., Rupnik Z., Smit Z. (2015). Analysis of metals with luster: Roman brass and silver. Nucl. Instrum. Methods Phys. Res. Sect. B Beam Interact. Mater. At..

[19] Ingo G.M., Riccucci C., Lavorgna M., Salzano de Luna M., Pascucci M., Di Carlo G. (2016). Surface investigation of naturally corroded gilded copper-based objects. Appl. Surf. Sci..

[20] Rezk R.A., Abdel Ghany N.A., Mostafa A.M. (2022). Laser-Assisted Method for Cleaning and Analysis of Archaeological Metallic Coins. Coatings.

[21] Drob A., Vasilache V., Bolohan N. (2021). The Interdisciplinary Approach of Some Middle Bronze Age Pottery from Eastern Romania. Appl. Sci..

[22] Gupta S., Sharma A.K., Agrawal D., Lanagan M.T., Sikora E., Singh I. (2023). Characterization of AZ31/HA Biodegradable Metal Matrix Composites Manufactured by Rapid Microwave Sintering. Materials.

[23] Zhang Y., Xiao Z., Meng X., Xiao L., Pei Y., Gan X. (2023). Experimental and Numerical Studies on Hot Compressive Deformation Behavior of a Cu–Ni–Sn–Mn–Zn Alloy. Materials.

[24] Farcas I.A., Dippong T., Petean I., Moldovan M., Filip M.R., Ciotlaus I., Tudoran L.B., Borodi G., Paltinean G.A., Pripon E. (2023). Material Evidence of Sediments Recovered from Ancient Amphorae Found at the Potaissa Roman Fortress. Materials.

[25] Rusca M., Rusu T., Avram S.E., Prodan D., Paltinean G.A., Filip M.R., Ciotlaus I., Pascuta P., Rusu T.A., Petean I. (2023). Physicochemical Assessment of the Road Vehicle Traffic Pollution Impact on the Urban Environment. Atmosphere.

[26] Chirila E., Gudea N., Cabuz I. (1970). Salajeni Hoard XVII—Century. Monetary Hoards in the Northen Transylvania between XVI–XVIII Centuries.

[27] Chirila E., Bajusz I. (1978). Monetary hoard of Verveghiu XVI–XVII centuries. Acta Musei Porolisensis.

[28] Pripon E. (2018). Medieval and Pre-Modern Monetary Hoards Discovered on the Area of Zalau City.

[29] Chirila E. (1970). Mineu monetary hoard XVII century. Monetary Hoards in the Northen Transylvania between XVI–XVIII Centuries.

[30] Chirila E., Lucacel V. (1965). Feudal hoard of Aghires XVI–XVII centuries. Stud. Univ. Babeș-Bolyai Ser. Hist..

[31] Moxon R., Xu Z., Chris-Okoro I., Cherono S., Kumar D. (2023). Determination and Calculations of Mercury Vapor Concentration and Energy Released from Freshly Condensed Dental Amalgams Having Various Copper Percentages within the Alloy. Materials.

[32] Barek J., Fischer J., Navratil T., Peckova K., Yosypchuk B. (2006). Silver Solid Amalgam Electrodes as Sensors for Chemical Carcinogens. Sensors.

[33] Liang P., Zhang J., Kong N., Li H., Zhu H. (2023). The Microstructure Characteristics Evolution of Bulk High-Purity Silver for High Relief Application. Metals.

[34] Ning Z., Wang Q., Zhao D., Pei W., Wen M. (2022). Structural Evolution of Bulk Silver during Cold Rolling and Annealing. Metals.

[35] Xu J., Gao J., Qin H., Liu Z., Zhu L., Geng H., Yao L., Zhao Z. (2022). Cu Nanowires and Nanoporous Ag Matrix Fabricated through Directional Solidification and Selective Dissolution of Ag–Cu Eutectic Alloys. Materials.

[36] Zuo X., Zhao C., Zhang L., Wang E. (2016). Influence of Growth Rate and Magnetic Field on Microstructure and Properties of Directionally Solidified Ag–Cu Eutectic Alloy. Materials.

[37] Riccucci C., Ingo G.M., Pascucci M., Staccioli M.P., Pierigè M.I., Albini M. (2022). Micro-structural and micro-chemical investigation of Samnite high-tin bronze cuirass discs: Ancient metallurgy in central Italy. Microchem. J..

[38] Ingo G.M., Riccucci C., Giuliani C., Faustoferri A., Pierigè I., Fierro G., Pascucci M., Albini M., Di Carlo G. (2019). Surface studies of patinas and metallurgical features of uncommon high-tin bronze artefacts from the Italic necropolises of ancient Abruzzo (Central Italy). Appl. Surf. Sci..

[39] Park J.S., Park C.W., Lee C.J. (2009). Implication of peritectic composition in historical high-tin bronze metallurgy. Mater. Charact..

[40] Petan A., Petean I., Paltinean G.A., Filip M.R., Borodi G., Tudoran L.B. (2023). Microstructural Investigation of Some Bronze Artifacts Discovered in a Dacian Site Using Non-Destructive Methods. Metals.

